# Integrating a pharmacist into the general practice environment: opinions of pharmacist’s, general practitioner’s, health care consumer’s, and practice manager’s

**DOI:** 10.1186/1472-6963-12-229

**Published:** 2012-08-01

**Authors:** Christopher Freeman, W Neil Cottrell, Greg Kyle, Ian Williams, Lisa Nissen

**Affiliations:** 1 School of Pharmacy, University of Queensland, Brisbane, Australia; 2 Camp Hill Healthcare Brisbane, Queensland, Australia; 3 UQ-Greenslopes Clinical School, Greenslopes Private Hospital, Brisbane, Australia; 4 Discipline of Pharmacy, University of Canberra, Canberra, Australia; 5 School of Medicine, University of Queensland, Brisbane, Australia

## Abstract

**Background:**

Pharmacists are viewed as highly trained yet underutilised and there is growing support to extend the role of the pharmacist within the primary health care sector. The integration of a pharmacist into a general practice medical centre is not a new concept however is a novel approach in Australia and evidence supporting this role is currently limited. This study aimed to describe the opinions of local stakeholders in South-East Queensland on the integration of a pharmacist into the Australian general practice environment.

**Methods:**

A sample of general practitioners, health care consumers, pharmacists and practice managers in South-East Queensland were invited to participate in focus groups or semi-structured interviews. Seeding questions common to all sessions were used to facilitate discussion. Sessions were audio recorded and transcribed verbatim. Leximancer software was used to qualitatively analyse responses.

**Results:**

A total of 58 participants took part in five focus groups and eighteen semi-structured interviews. Concepts relating to six themes based on the seeding questions were identified. These included positively viewed roles such as medication reviews and prescribing, negatively viewed roles such as dispensing and diagnosing, barriers to pharmacist integration such as medical culture and remuneration, facilitators to pharmacist integration such as remuneration and training, benefits of integration such as access to the patient’s medical file, and potential funding models.

**Conclusions:**

These findings and future research may aid the development of a new model of integrated primary health care services involving pharmacist practitioners.

## Background

Pharmacists have traditionally performed technical roles such as dispensing and compounding of medications. In more recent times the focus has shifted to strengthening the role of medication information provision as well as improving the quality use of medication through activities such as medication management reviews and chronic disease management programs [1]. Australia, like many developed nations, is undergoing fundamental changes to the health care system [2]. With the current financial stressors, the Australian government is looking to improve value for their investment in health care [2]. Pharmacists are viewed as highly trained yet underutilised professionals in the Australian health care system and there is growing support to extend the role of the pharmacist within the primary health care sector [3]. In the 2010 – 11 budget, the Australian government committed a further $AUS370 million to the development of the GP [general practice] Superclinic program with many clinics incorporating pharmacist services [4]. These clinics intend to bring together general practitioners (GPs), visiting specialists, nurses, and allied health professionals to deliver health care suited to the needs of the surrounding community. This creates opportunity to explore new models of practice to further utilise the pharmacist in the primary care setting. The integration of a pharmacist into a general practice medical centre is not a new concept [5] however is a novel approach in Australia and evidence supporting this role is currently limited [6].

International research on this topic has focused on the views of GPs, health care consumers, and pharmacists, once the pharmacist was already integrated into the general practice medical centre [7-15]. Responses from GPs were positive recognising the potential benefit of integrating a pharmacist into the medical team with the recognised benefits perceived by the GPs increasing over time [7,9,12,14,15]. Research involving health care consumers found that patients are generally supportive of a pharmacist involvement in non dispensing roles [10]. However, some health care consumers found it difficult to foresee the benefits potentially offered by a pharmacist in the general practice medical centre setting largely due to being unfamiliar with the clinical roles of a pharmacist [13]. The experiences expressed by pharmacists in qualitative reports appear common to starting any new job including getting to know co-workers and orientation to the practice environment [8,11,16,17]. Pharmacists described a non linear process, rather like a rollercoaster, with successes one day and drawbacks the next [8]. The results demonstrate that an appropriate set of skills are required for a pharmacist to initially integrate into this environment. These include the ability to overcome emotional challenges such as perceived professional inadequacy and developing clinical aptitude for tasks such as patient assessment [8,16].

Foundational research on this topic in the literature is sparse. Integrating a pharmacist into the Australian general practice environment is a novel concept and requires a new model of practice to be developed. Investigating the views and opinions of key stakeholders on the practice model is potentially crucial to the model’s success [18]. This research would help identify which services would be of value and identify primary barriers and facilitators to service provision as identified by stakeholders. Opinions obtained through qualitative inquiry from different perspectives may foster inter-professional development of the potential model. This may then facilitate a greater opportunity for the model to be implemented successfully [18].

The aim of this study was to describe the opinions of general practitioners, health care consumers, pharmacists, and practice managers in South-East Queensland on the integration of a pharmacist into the Australian general practice environment.

## Methods

Content analysis of focus groups and semi-structured interviews was utilised to explore the opinions of pharmacists, general practitioners, health care consumers, and practice managers. Semi-structured interviews were offered in addition to the focus groups to provide the participants with a choice, where public discussion of business practices was not desirable. This method allowed a greater depth of investigation in opinions and could inform a wider investigation through the use of other methods such as a national survey.

A convenience sample of pharmacists, general practitioners, health care consumers, and practice managers in South-East Queensland was sort to participate in focus groups or semi-structured interviews. Participants were identified through advertisement via local divisions of general practice and professional networks (contacts in relevant professional organisations) already established by the investigators.

Immediately prior to each focus group/semi-structured interview, participants were asked to complete a short (anonymous) questionnaire to provide data related to any experience they have recently (last 12 months) had with pharmacy cognitive services (e.g. such as a home medicines review (HMR)). A questionnaire was chosen to avoid personal information being discussed in the focus group and provided basic age and gender demographics.

Focus groups and interviews were conducted by two of the investigators (CF and GK) in face to face sessions. Seeding questions common to all the focus groups and semi-structured interviews were used to facilitate discussion, to help maintain consistency, and to reduce any potential for interviewer bias. The questions were grouped into themes (positively viewed roles, negatively viewed roles, barriers to integration, facilitators to integration, benefits of integration, and remuneration) to allow for in-depth analysis. Themes were based on topics the investigators determined would best contribute to fulfilling the aim. Dissenting or new themes were also explored when raised by the participants to comprehensively explore the topic. The focus groups and interviews were continued until a saturation of themes was established.

All focus groups and interviews were audio recorded and transcribed verbatim by an independent contractor. Transcripts were then checked for accuracy by one of the investigators (CF). Leximancer (UQ, version 3.5) software was used to analyse the transcripts. Leximancer is a text mining software package which performs an automatic analysis of the text through eliciting emergent concepts and is based on Bayesian decision theory [19]. The term “concept” when used in the context of Leximancer can be defined as a common single or compound word which correlates commonly with other words in a body of text. This methodology of textual analysis has previously been validated and is becoming more widely used in the health care setting [20,21] (Additional file 1).

Leximancer identifies the prevalence of a concept and concept correlation within the transcripts to formulate a co-occurrence matrix (a mathematical calculation that allows the prevalence of co-occurrence of all concepts against all others to be explicitly stored). The information is displayed by means of a conceptual map that provides a bird’s eye view of the material, representing the main concepts contained within the text and information about how they are related [22]. Concepts are represented as dots on the concept map. The more commonly a concept correlates within the transcripts, the larger the dot becomes, indicating greater importance of the concept. Concepts which appear in a similar context (same sentence block (set at 2 sentences)) within the transcripts are clustered closer together. Hence, related concepts are located close together on the concept map while the opposite is true for unrelated concepts. Once concepts are identified, Leximancer permits further exploration by allowing the user to view the text in which the concepts occur. Representative quotes used in the results section of this paper were derived through this process.

Leximancer software also allows “tags” to be applied to separate transcripts which then are displayed on the concept map. A tag is the name of the document file or folder in which the transcripts are stored. Tags are treated the same way as concepts in Leximancer, so the set of rules which applies between concepts also occurs between tags and concepts. Concepts which commonly correlate in the transcript to which a tag has been applied are clustered closer to each other and to the tag. Compared with other qualitative methods (*NVivo*, *NUDist*), Leximancer analyses are based entirely within the text, rather than on researcher-driven interpretive coding.

The Leximancer concept analysis can then be interpreted to extract meaning from the concept relationships. This provides greater transparency in qualitative analysis as the base (automated) analysis is presented as is the interpretation. A drawback of researcher-driven interpretive coding is a lack of transparency in the coding phase.

Transcripts from all focus groups and semi-structured interviews were analysed through Leximancer to develop an overall view of the data. The transcripts were then reorganised into groups of respondents (*pharmacist*, *GPs*, *health care consumers*, *practice managers*, and *mixed pharmacist and GP* group) and then further subdivided into themes based on the seeding questions (positively viewed roles, negatively viewed roles, barriers to integration, facilitators to integration, benefits of integration, and remuneration). For example, all responses given by pharmacists to the seeding questions themed around positively viewed roles were compiled into one document. The reorganised transcripts were then individually analysed through Leximancer and the emerging concepts presented in table format. As Leximacer produces concepts as a single term, such as *medication*, some concepts were joined to produce new, more meaningful concepts. For example, the concepts *medication* and *review* were joined to create new concept *medication review*. This allows for a more explicit representation of the participants opinions from the above identified themes.

Ethical clearance was obtained from the University of Queensland Human Medical Research Ethics Committee and each participant provided written informed consent.

## Results

A total of 58 participants took part in five focus groups and eighteen semi-structured interviews. Table 1 shows the style of interview used, the number of participants, the group in which the participant’s belong, and participant demographic information.

**Table 1:** The style of interview used, the number of participants, and the participant’s group and demographic information

**Group**	**Style of Interview**	**Number of participants**	**Age Range**	**Female Gender**	**Previous experience with pharmacist conduct cognitive services (i.e. HMR)**
**Pharmacists**	Focus Group 1	6	23 – 65 years	52 %	68 %
	Focus Group 2	9			
	Semi-structured Interviews	10			
**General Practitioners**	Semi-structured Interviews	4	46 – 65 years	25 %	75 %
**Health Care Consumers**	Focus Group 1	8	76 – 85 years	87 %	17 %
	Focus Group 2	10			
**Practice Managers**	Semi-structured Interviews	4	46 – 55 years	100 %	*
**Mixed Group (GPs and Pharmacists)**	Focus Group	4 General Practitioners 3 Pharmacists	26 – 55 years	71 %	100 %

*Practice managers indicated that they did not directly participate in these services however GPs and health care consumers from their medical centres had.

**Figure 1:**
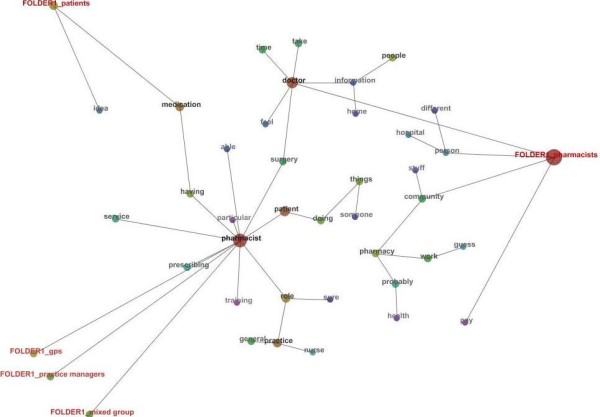
**Concept Map produced from Leximancer of Focus Groups and Semi Structured Interviews.** Folder = transcripts grouped by type of participant. The colour of the concept dots represents the frequency which the concept occurred. The red spectrum indicates high concept occurrence and blue spectrum indicates low concept occurrence.

The concept map generated by Leximancer of the combined transcripts is represented in Figure 1. Thirty-seven concepts were automatically identified with *pharmacist*, *doctor*, *medication*, *patient*, *practice*, and *role* the most repeatedly identified. Four tags (*pharmacists*, *GPs*, health care consumers (*patients*), *practice managers*, and *mixed group* (pharmacists and GPs)) are also displayed on the concept map indicating the different groups of participants.

The concept map (Figure 1) shows that the concept *pharmacist* was central in all interviewee’s responses. The concepts of *general*, *having*, *surgery*, and *role* commonly co-occurred with the concept *pharmacist*, indicating that the “role of a pharmacist in a general practice surgery” was discussed repeatedly.

*“So I certainly agree I see a role for the pharmacist giving lifestyle advice and that sort of stuff within in a practice. I think that'd be useful“.* (General Practitioner)"

The group tags of *GPs*, *practice managers*, and the *mixed group* are all clustered together suggesting that the opinions of these groups are closely aligned. The concept map shows that interviews from these three groups focus around the concept *pharmacist*. This is in contrast to the health care consumers and pharmacists tags. The concept *pharmacist* appears disconnected to the *pharmacists* group in the concept map however has occurred due to the pharmacists referring to themselves as “I” or “we” rather than using the term “pharmacist”. Furthermore, the *pharmacists* group is linked to the concept *pharmacist* via the concepts *doctor* and *surgery*.

The pharmacists tag is much larger than the other group tags resulting from a larger amount of content from more participants (Table 1). Closely connected to the tag for the pharmacist group was the concepts *community*, *hospital*, and *person*, indicating that pharmacists as a group discussed the transfer of a person/patient from hospital into the community and the potential role which a practice pharmacist may have.

*“I would see the role as beneficial being a facilitator role, maybe even a link between the community pharmacy and the GP or the hospital”.* (Community Pharmacy Proprietor)"

The concept medication is most closely connected to the health care consumer (patient) group tag. This suggests that health care consumers frequently discussed the impact of this new model on their medications.

*“I think that it's [pharmacist located within the medical centre] a good idea for people that are nervous and apprehensive about their medication and they'd like it explained thoroughly”.* (Health Care Consumer)"

The transcripts were reorganised into the different groups of participants and then further subdivided into themes based on the seeding questions (positively viewed roles, negatively viewed roles, barriers to integration, facilitators to integration, benefits of integration, and remuneration). The reorganised transcripts were then individually analysed through Leximancer and the emerging concepts presented in a table format. (Table 2)

**Table 2:** Concepts derived by Leximancer from interviews divided into respondent groups and themes

**General Practitioners**	**Pharmacists**	**Health Care Consumers**	**Practice Managers**	**Mixed Pharmacist and GP Group**
**Positively viewed roles**				
Medication review	Medication review	Medication review	Medication review	Medication review
Medication information	Medication reconciliation/history taking	Medication information/counselling	Medication counselling	Medication information
Education to patients	Education	Patient advocate to GP	Education and Drug Information	Patient education/Patient medication profile print outs
	(GPs and Patients)			
Quality prescribing initiatives	Specialty clinics	Medication profile print outs	Medication Reconciliation	Cost savings on medications
Education to GPs	Prescribing	Prescribing (script renewal)	Repeat prescribing	Repeat prescribing
**Negatively viewed roles**				
Dispensing	Dispensing	Dispensing	Dispensing	Ordering pathology
Prescribing	Prescribing	Prescribing	Prescribing	Prescribing
Diagnosing	Diagnosing	Physical examination/diagnosing	Diagnosis	Diagnosing
	Procedural tasks		Immunisations	Routine GP services
**Barriers to integration**				
Remuneration	Remuneration	Remuneration	Funding	Funding
Size of practice	Medical culture/“Turf wars”	Reluctance from GPs	Turf wars	Size of the practice
Lack of space	Operational/logistical issues	Physical space	Size of the practice/available space	Operational
Preconceptions of pharmacist roles	Experience of the pharmacist		Logistical issues	
**Facilitators to integration**				
Remuneration	Remuneration	Remuneration	Funding	Funding
Training of the pharmacist	Training of the pharmacist	Support of GPs	Additional training of pharmacist	Changes in legislation/business rules for services
Defined scope of practice	Education on role to medical profession	Promoting services to community	Education to GPs on benefits	
Administrative support		Administration support	Education to patients on benefits	
**Potential benefits of integration**				
Increased access and communication	Access to patient medical file	Access to medical notes and GPs	Access to patient medical file	Access to patients record
Increased rapport	Privacy	Privacy	Increased privacy	Privacy
Pharmacist seen as independent	Dedicated time for services	Dedicated time to spend with patient	Increased rapport and communication between pharmacist and GP	Dedicated time
Increased patient acceptance of pharmacist services	Increased rapport and communication with GP	Closer working relationship with GPs	Enhanced coordination of services	Less commercial influences
		Reduce GP workload	Continuity of care	
**Method of remuneration**				
Government	Government	Government	Government	Government/DVA
	Patient	Patient	Patient co-payment	
	Medical Centre	Medical centre		
	Health Insurance			

DVA = Department of Veteran Affairs.

Concepts are not ranked.

Medication reviews, medication information, and education were universally considered as positive roles for a pharmacist to perform within the general practice environment. Prescribing conducted by the pharmacist in this setting was seen both as a positive and negative role by pharmacists, health care consumers, practice managers, and the mixed group, whereas the GPs viewed pharmacist prescribing negatively. The concept map (Figure 1) presents the concept *prescribing* directly connected to the concept *pharmacist* with both concepts closest to the GPs, mixed group, and practice managers groups. This also indicates that the previously stated groups discussed this topic more often within the interviews. Pharmacists performing diagnosis within the general practice environment was universally reported as a negatively viewed role.

*“I would assume that they [pharmacists] wouldn't take on roles that encroached on the professional roles of nurses for instances in applying wound dressings or giving advice beyond their scope of practice – you know, diagnosis and management of complex conditions would be taking on a challenging task I would have thought”.* (General Practitioner)"

When the health care consumer group viewed pharmacist prescribing as a positive; the focus was on the renewal of prescriptions. They generally did not see the pharmacist prescribing more or less accurately or efficaciously than the GP, rather the patient group discussed this service as one which might save them money or time (not having to pay or wait for the GP).

“*I do agree with that [pharmacist prescribing] because I'm a case in point on this - that many times I go to the doctors, not because I'm feeling ill, but because I've reached the stage where I need a couple of prescriptions. You have to sit for maybe an hour, hour and half, two hours to get repeat prescriptions. You're in and out in three to four minutes*.” (Health care consumer)"

While this was the case generally, some health care consumers did report that pharmacists have a great depth of medication knowledge and might be a suitable alternative to a GP prescribing.

“*I think it's a bit like we're saying the pharmacist knows medicines best but the doctor knows bodies best and diseases best*.” (Health care consumer)"

Figure 1 shows the path connecting the concept *pharmacist* to the concept *nurse* includes the concepts *role* and [*general*] *practice*. This indicates that the participants discussed the potential role of a pharmacist in this practice setting and how that may impact on the role of the practice nurse.

Table 2 also highlights some important differences in the roles identified between the groups of participants. The GPs identified quality prescribing activities such as clinical audits as a potential role for a practice based pharmacist and was not discussed by the other groups. The pharmacist group discussed the potential for their involvement in specialty clinics particularly focusing on chronic diseases such as COPD and diabetes. The health care consumers discussed how the pharmacist could act as an advocate for them, a concept not identified by any of health professional groups.

The concepts of providing remuneration and lack of remuneration were viewed by the interviewee’s as a facilitator and a barrier to service provision respectively. Medical culture/”turf wars” or preconceptions was a common barrier reported by most groups. Additional training of the pharmacist was considered a potential facilitator to service provision by the GP, pharmacist, and practice manager groups. The concept map (Figure 1) also shows that the concepts *pharmacist*, *training*, and *role* are closely clustered together.

*“I think as a pharmacist you can’t rest on your Bachelor of Pharmacy. I think thatit really needs extra training for a lot of the functioning’s within the GP clinic ....”* (Accredited Pharmacist)"

The health care consumer and the practice manager groups identified that promotion of services provided by the pharmacist could help the facilitation into the general practice setting and could increase demand for services. This was not discussed by the other groups of participants.

The groups saw the potential advantage of the medication review service as well as the other services identified in Table 2, being integrative as well as collaborative. They highlighted that integration would serve as the point of difference from the existing services being offered externally by pharmacists. These advantages are outlined in Table 2 (potential benefits to integration). These may include but are not limited to, having access to the shared patient medical file, improved rapport between the GPs and the pharmacist, and increased communication and collaboration between health care providers. Figure 1 shows the concepts *pharmacist* and *doctor* linked through the concept *surgery*. This suggests that participant’s opinions were based on the two health professionals integrated in one physical location (surgery – meaning medical centre) providing a vital link for service provision and communication. Access to the patient’s medical file and increased rapport/communication between the pharmacist and GP were also commonly considered benefits to integrating pharmacist services within the general practice environment. Increased privacy was viewed as a benefit by all groups except the GP group.

Some form of government remuneration for pharmacist services was collectively reported by all groups as a funding model. Although the concept map (Figure 1) sparsely depicts the concepts *pay* and *service*, they occurred commonly together in the transcripts indicating frequent conversation on how pharmacist’s services could to be remunerated.

## Discussion

The focus groups and semi-structured interviews outlined above give a unique insight into the opinions and views of pharmacists, general practitioners, health care consumers, and practice managers around integrating pharmacists into the Australian general practice environment.

When the participants were asked to discuss what roles they would perceive a pharmacist conducting within this setting, the responses resembled services already offered by pharmacists externally. This is highlighted by all groups of participants reporting medication review as a primary service.

Medication reviews conducted by a pharmacist within the general practice environment have already been established in Canada and the USA [23,24]. The signing of the Fifth Community Pharmacy Agreement (5CPA) in May 2010 has seen a change in the business rules for the home medicines review (HMR) program, allowing GPs to directly refer to an accredited pharmacist of the patient’s choice [25]. This may potentially provide a source of remuneration for a pharmacist to conduct medication reviews based at a general practice medical centre.

Our results indicate a division of opinions regarding the potential for pharmacists to prescribe medications. This was demonstrated both within and between groups of participants. Internationally, pharmacists have obtained authority to prescribe and some pharmacists are prescribing within the primary care/general practice environment [26]. The health care consumer groups in our study identified greater accessibility and a reduction of costs as benefits to pharmacist prescribing. This may be an extension of the health care consumer’s perception on retail pharmacy where they generally do not pay for professional services offered by pharmacists. Other health care consumers recognised attributes such as medication knowledge as benefits to pharmacist prescribing. Both of these sentiments have been reflected in Australian literature which has explored patient’s views on pharmacist prescribing [27].

Pharmacist participation within primary care speciality clinics such as chronic disease clinics (COPD) or therapeutic clinics (anticoagulation) was seen as a positive role by the pharmacist group however not by the other groups of participants. Internationally pharmacists have been valued members of specialty clinics [28] however in Australia research in this area is confined to community pharmacies [29].

The health care consumer group viewed a potential role for a pharmacist in this setting as an advocate and was not discussed by other groups of participants. Health consumer advocacy has long been described as a role of the nursing profession [30] however might be an underappreciated role for the pharmacist particularly in this setting.

Privacy, access to the patient’s medical file, and increased rapport and communication between the GP and the pharmacist were reported as potential benefits to integrating a pharmacist. These opinions are supported by previous research which indicates that having a pharmacist integrated into this environment increases rapport between health professionals, enhances information exchange and facilitates communication [7,31-35].

The majority of community pharmacies in Australia have limited space or scope available to provide private consultation. Having a pharmacist integrated into the medical centre could take advantage of the existing infrastructure to enable consultation to be held in a private space. Further, the literature reports that patient indifference to pharmaceutical care was diminished by having a pharmacist integrated into the medical centre compared to a community pharmacy [7].

Additional training of the pharmacist was viewed by the GPs, pharmacists, and practice manager groups as a potential facilitator to the development of this model. This was also described in a pilot study from Canada, where training and mentoring were reported as facilitators [8]. Each pharmacist, prior to participating in the pilot, received training from a family practice simulator module and was assigned a mentor who assisted with skill development, role modelling and coaching, and emotional needs of the pharmacist.

Medical culture or “turf wars” were described as a potential barrier by the pharmacists, health care consumers and practice manager groups, however was not discussed by the GP group. This may indicate that this barrier is only perceived by the groups which mentioned this. The potential roles the GP group highlighted for the pharmacist to provide in this setting (Table 2) can be classified as “value added services” and do not duplicate the current role of a general practitioner. This may appear less threatening to the GP group of participants. In a review of the evidence investigating the support for skill mix changes between GPs, pharmacists, and nurses, Dennis et al. emphasized the importance *to support an effective skill mix that ensures the task complements rather than duplicates the work of the GP*[36].

A lack of appropriate or sustainable remuneration for the pharmacist was collectively seen as the largest barrier to service provision by all groups of participants. This is well described in the literature which indicates that the barrier is inherently linked to the structure of the health care system [7,37]. Australia’s health care system is dominated by fee for service arrangements which has many limitations such as narrowing the scope of services available and the potential for provider abuse. Participants in our study agreed that a mix of government and private funding was an appropriate model of remuneration.

The interpretation of the results may be limited by the small numbers of participants and the confined area the participants were recruited from. However as a general rule primary health care policy and practice is uniform nationwide. To help maintain consistency, and to reduce any potential for interviewer bias seeding questions common to all the focus groups and semi-structured interviews were used to facilitate discussion. As with all forms of qualitative research tools, Leximancer, despite its automation, still has subjective elements which rely on human interpretation to elicit meaning from the data. However, the presentation of automatically generated results and the interpretation adds transparency to this process. The convenience nature of the sample has the potential to positively bias the results.

## Conclusions

Focus groups and semi-structured interviews conducted with general practitioners, pharmacists, healthcare consumers, and practice managers have highlighted potential roles for a pharmacist practicing within a general practice medical centre. Facilitators and barriers to service provision, the potential benefits of integrating a pharmacist into the general practice setting, and possible remuneration methods were also identified. These findings will aid the development of a new model of integrated primary health care involving pharmacist practitioners. A larger study which seeks the opinions of key informants on a national scale is required to confirm these results.

## Competing interests

The authors declare no competing interests.

## Authors’ contributions

CF and GK facilitated the focus groups and semi-structured interviews. CF checked the transcriptions for accuracy, completed the analysis of the transcripts and drafted the manuscript. All authors conceived the study and participated in its design and read and approved the final manuscript.

## Pre-publication history

The pre-publication history for this paper can be accessed here:

http://www.biomedcentral.com/1472-6963/12/229/prepub

## Supplementary Material

Additional file 1 Interview Guide. Click here for file
