# Supplementary material for: Integrating a pharmacist into the general practice environment: opinions of pharmacist’s, general practitioner’s, health care consumer’s, and practice manager’s
Source: BMC Health Serv Res. 2012 Aug 1;12:229. doi: 10.1186/1472-6963-12-229 (PMC3444319; doi:10.1186/1472-6963-12-229)
Supplement: Additional file 1 — Interview Guide. [file 1472-6963-12-229-S1.doc]

# Interview Guide

## General Practitioners, Pharmacists, and Practice Managers

1. In general do you believe it is appropriate to have a (non dispensing) pharmacist integrated into a general practice environment?
   1. If so, why?; If not why not?
2. Specifically what roles would you perceive a pharmacist performing in a general practice environment?
3. Specifically what roles would you **not** perceive a pharmacist performing in a general practice environment?
4. Are there any current pharmacist services provided outside the general practice environment that could be adapted to be performed within a general practice environment?
5. Are there any roles/activities which a pharmacist does not currently perform that would be beneficial in a general practice environment?
6. What do you think are the **barriers** to having a pharmacist integrated into a general practice environment?
7. What do you think are the **enablers** to having a pharmacist integrated into a general practice environment?
8. What do you perceive as the benefits of having a pharmacist co-located within the general practice environment?
9. How do you think services conducted by an integrated pharmacist should be funded?

## Seeding Questions to Health Care Consumers

1. In general do you believe it is appropriate to have a pharmacist (not dispensing medications) working within a general medical practice?
2. Specifically what roles would you perceive a pharmacist performing in a general practice environment?
3. Specifically what roles would you **not** perceive a pharmacist performing in a general practice environment?
4. Are there any current pharmacist services provided outside the general practice environment that could be adapted to be performed within a general practice environment?
5. Are there any roles/activities which a pharmacist does not currently perform that would be beneficial in a general practice environment?
6. Do you think there is anything that would **prevent** having a pharmacist integrated into a general medical practice environment?
7. Do you think that there is anything that would **make it possible** to have a pharmacist integrated into a general practice environment?
8. What do you perceive as the benefits of having a pharmacist co-located within the general practice environment?
9. How do you think services conducted by an integrated pharmacist should be funded?
   1. Would you be willing to pay for pharmacist services conducted within a medical centre environment?
